# Scoping Review of Adult Emergency Department Discharge Interventions

**DOI:** 10.5811/westjem.35264

**Published:** 2025-07-13

**Authors:** Mary-Kate Gorlick, Shriman Balasubramanian, Gregory Han, Andy Hickner, Pranita Talukder, Peter AD Steel, Lynn Jiang

**Affiliations:** * UT Health Houston, Department of Emergency Medicine, Houston, Texas; † NewYork-Presbyterian Hospital, Department of Emergency Medicine, New York, New York; ‡ Weill Cornell Medicine, Samuel J. Wood Library & C.V. Starr Biomedical Information Center, New York, New York

## Abstract

**Introduction:**

The discharge process is a crucial component of the emergency department (ED) encounter, with poor discharge quality often leading to negative patient outcomes. While numerous interventions have been implemented to improve this process, a comprehensive review of these interventions has not been conducted. This study provides a scoping, summative review of adult ED discharge interventions to date, evaluating the literature for potential best practices and future directions.

**Methods:**

We conducted a scoping review of published literature on MEDLINE ALL (Ovid), Embase (Ovid), the Cochrane Central Register of Controlled Trials (Wiley), and CINAHL (EBSCOhost) on February 7, 2023, for articles reporting on ED-based discharge interventions. We excluded the following: studies involving pediatric patient populations; discharge from non-ED settings; in-ED risk screening and/or case management as the primary intervention; interventions occurring mostly after the ED encounter (even if initiated at time of discharge); and studies not written in English.

**Results:**

The initial electronic database search yielded 3,842 unique titles and abstracts. After applying inclusion/exclusion criteria at various screening stages, we included 100 papers and abstracts in the final review. These studies, published between 2003 – 2023, predominantly originated from the US (66%). Using narrative synthesis, we summarized ED discharge intervention themes to form seven concept subgroups by consensus: mode of discharge; additional resource provision; addition of a discharge coordinator; follow-up assistance; pharmaceutical intervention; patient-centered education; and clinician/discharger-centered education. Effective strategies included enhanced discharge discussions and education by dedicated personnel, structured discharge checklists, and delivery of instructions at an appropriate reading level. However, because few studies have examined long-term patient-centered outcomes, such as ED return visits, hospitalizations, and mortality, cost-benefit analysis for interventions is lacking. Furthermore, the experiences of vulnerable populations who have limited-English proficiency are under-represented in current attempts to innovate ED discharge.

**Conclusion:**

We found that interventions aimed at improving patient comprehension of discharge instructions were the most frequently studied and had the greatest impact on patient outcomes. This review highlights promising directions for patient-centered innovation; it also underscores the need for more research to optimize the adult ED discharge process and warrants a call to action.

## INTRODUCTION

Discharge is the release of a patient following the formal completion of a healthcare evaluation, procedure, or course of treatment. The emergency department (ED) discharge process provides post-ED care information to patients by the ED care team, typically in both verbal and written form or electronic documents. This final step in the ED encounter gives emergency clinicians the chance to conceptually “land the plane,” completing an encounter that frequently includes multiple diagnostic and therapeutic interventions, as well as complex medical decision-making and risk stratification. The ideal structured, inclusive, and comprehensive approach to “closing” the ED encounter would ensure a patient understands what transpired during their ED evaluation and empower them with the appropriate information and comprehension of their post-ED care plan. This is distinct from care coordination, which organizes and facilitates longitudinal patient care and communication among all participants in patients’ healthcare needs; however, the ED discharge process is one step in that coordination continuum.

The Agency for Healthcare Research and Quality (AHRQ) along with emergency medicine (EM) organizations (Academy of Emergency Physicians and Society of Academic Emergency Medicine) recognize the significance of a high-quality ED discharge.^1^–^3^ These agencies have developed summative guidelines for optimal discharge processes that include discussion of the patient’s ED diagnosis; expected course of illness; home care instructions; medication use; follow-up care; reasons to return to the ED; and opportunities for the patient to ask questions and/or confirm understanding.

Despite this consensus, ED space, resources, and time constraints frequently challenge patient-centered processes,^4^–^7^ with research demonstrating deficiencies in current ED discharge. Quality of discharge instruction content is frequently variable, often missing key components.^8^ Moreover, as many as 50% of US adults have low health literacy, leading to comprehension issues when content delivery is not at the appropriate reading level or in the appropriate language.^9^ Additionally, patients are often given insufficient time to ask questions, and comprehension is rarely assessed.^10^

Inadequate ED discharge quality can lead to poor outcomes; poor comprehension of and low adherence to post-ED care instructions contribute to high ED return rates and adverse events.^11^–^13^ Limited comprehension of ED discharge instructions has also been shown to positively correlate with low post-ED care compliance.^14^ Encouragingly, recent efforts to improve the quality of ED discharge has significantly reduced the odds of ED return visits and increased compliance with taking newly prescribed medications.^15^

The increasing focus on health equity in EM has led to extensive work on transitions of care and integrated health systems. Despite the importance of the ED discharge process, discharge instructions are often not of consistently high quality, and these limitations may lead to poor patient outcomes. To date, no scoping review has examined the breadth of adult ED interventions with the goal of improving this process. Previously published reviews have examined ED discharge risk-stratification^16^ and the relationship between the way discharge instructions are conveyed and patient comprehension.^17^ The AHRQ commissioned an extensive environmental overview of the ED discharge process, focusing on risk factors leading to poor-quality discharge.^18^ That overview included pediatric populations as well as care coordination and after-ED care. In this study we provide a scoping, summative review of adult ED discharge interventions, evaluating the literature for potential best practices and future directions.

**Population Health Research Capsule**

What do we already know about this issue?

*Poor ED discharge quality leads to adverse outcomes. While interventions exist, no comprehensive review has evaluated their effectiveness*.

What was the research question?

*What adult ED discharge interventions exist, and which are most effective?*

What was the major finding of the study?

*We analyzed 100 studies, categorizing ED discharge interventions into 7 themes. Most focused on patient comprehension, but few assessed long-term outcomes*.

How does this improve population health?

*This review highlights gaps in research on discharge interventions and could guide future efforts to develop evidence-based strategies for better patient outcomes*.

## METHODS

We constructed this scoping review using the Preferred Reporting Items for Systematic Reviews and Meta-Analysis – Scoping Reviews (PRISMA-ScR) guidelines.^19^ The PRISMA checklist can be found in Supplement 1. We searched MEDLINE ALL (Ovid), Embase (Ovid), the Cochrane Central Register of Controlled Trials (Wiley), and CINAHL (EBSCOhost), limiting our search to articles published from 2000 to February 7, 2023 (the date we conducted our search). The year 2000 was chosen to reflect the current state of practices, including technology-based interventions. We imported the de-duplicated results using *Covidence systematic review software* (Veritas Health Innovation, Melbourne, Australia), focusing on two concepts: discharge from the ED, and a list of specific discharge interventions. Searches incorporated both free-text keywords and, where available, controlled vocabulary; proximity operators were incorporated to retrieve variations of phrases. Where appropriate, controlled vocabulary terms were “exploded,” a technique that expands the search results. Further detail, including search strategies for all databases, can be found in the Supplementary Material and on the Open Science Framework website at https://osf.io/rtbfk.^20^

MKG, GH, and SB independently screened the imported titles and abstracts using Covidence. Discrepancies were resolved by consensus, with PS and LJ serving as tiebreakers. All citations were reviewed against predefined inclusion/exclusion criteria. Inclusion criteria were studies reporting on ED-based discharge interventions. We excluded non-English language articles. Additional exclusion criteria were as follows: pediatric (<18 years of age) patient population; discharge from inpatient, outpatient, non-clinical, or other non-ED setting; interventions including in-ED risk screening and/or case management as the primary intervention; and interventions where the majority of the primary intervention occurred after the ED discharge process, even if the intervention was initiated at discharge. Our final exclusion criteria enabled us to focus specifically on interventions occurring at time of discharge, as opposed to those in which the primary intervention occurred after the patient left the ED or earlier in their ED evaluation course.

A full-text review followed the initial title and abstract screening phase. Studies were selected for inclusion using the criteria outlined above. MKG, GH and SB extracted data using Covidence. Using the narrative synthesis approach described by Snilstveit et al,^21^ we grouped articles with similar ED interventions thematically. This methodology has been used in other ED-based scoping reviews.^22^–^24^

## RESULTS

See the figure for study selection workflow. The initial electronic database search yielded 3,842 unique titles and abstracts. Of these, 278 appeared to meet the inclusion criteria and were imported for full-text review. Of these, we included 100 papers and abstracts published between 2003–2023; 66% were published in the US.

Using narrative synthesis as described by Snilstveit et al, we summarized ED discharge intervention themes to seven concept subgroups by consensus (MKG, GH, SB, PS, LJ). The table lists the subgroups and their descriptions. Some studies were included in more than one subgroup.

### Mode of Discharge

Of 25 studies that examined mode of discharge, 18 (72%) were full-text studies. The most common study types were quasi-experimental (12/25, 48%) or randomized controlled trials (RCT) (9/25, 36%). A large proportion (13/25, 52%) investigated standardized discharge instructions. Standardization can partially automize writing discharge instructions, saving significant time, and can lead to improved quality.^12^,^25^,^26^ Interventions included creating a list of symptoms that should prompt re-evaluation, as well as simplifying 27–30 and creating templated discharge instructions.^31^–^33^ One study implemented a new template to encourage inclusion of ED diagnosis, follow-up care plan, and key results, which led to nearly universal comprehensive documentation including these important discharge aspects.

As familiarity with technology continues to increase,^34^ smartphones have become an important tool for delivery of discharge instructions due to the ease by which information and/or media can be relayed to patients.^35^–^37^ Several (10/25, 40%) papers investigated the use of electronic adjuncts for discharge instructions. Results showed patients preferred electronic delivery^38^–^42^ and appreciated video supplementation to the discharge instructions. Two studies examined the use of electronic delivery of patients’ discharge instructions to primary care clinicians, demonstrating an additional mode of discharge delivery leveraging technology.

The computerization of healthcare documentation improves quality of instructions and makes quality easier to achieve. These studies demonstrate novel ways to effectively streamline the process of writing quality discharge instructions and enhance their delivery with adjunctive media, as well as improve communication to primary care clinicians, However, those studies do not evaluate the effectiveness of these interventions on patient satisfaction, ED revisit rates, and follow-up rates.

### Providing Additional Resources

Three studies included an additional resource to assist in discharge. All were full-text studies with two prospective studies and one cross-sectional. Two focused on vulnerable patient populations. One study focused on elderly patients, leveraging discharge nurses and an already well-validated assessment tool to determine out-of-hospital resource needs.^43^ The nurse then provided these resources, making relevant referrals to home care services, outpatient clinics, community centers, and/or making arrangements with patients’ families. The second study involved county hospital volunteers who provided discharged patients with an educational intervention, medications, and follow-up appointment review, as well as individually tailored information for social and medical resources, including prescription discounts, low-cost clinics, rental assistance programs, and subsidized transportation.^44^ The interventions in both studies resulted in reduced ED return visits. The third study focused on ED patients with acute lower back pain, providing in-ED physical therapy evaluation and discharge exercise recommendations.^45^ Results from this study, however, did not show significant at-home compliance with the exercises.

Overall, while few studies incorporated additional resources and there was variety in study designs and outcomes examined, results show that it may be helpful to have personnel dedicated to particular patient subgroups to help coordinate care, follow-up, and comprehensive needs assessments. Finances may be an implementation barrier, as additional resources tend to require personnel, which increases costs. None of the studies addressed a cost-benefit analysis.

### Adding A Discharge Coordinator

Inclusion criteria that focused on discharge coordination interventions were aimed at improving the discharge *process*. Excluded were care-transition interventions that included in-ED risk screening and/or case management as the primary intervention. Seven studies, conducted in the US and interationally, met these criteria. There was variability in study type including descriptive methodological, quasi-experimental, case control, cohort, and a single RCT.

Five of seven studies (71%) implemented a dedicated nurse to coordinate patient discharge^46^–^49^; the nurse reinforced the discharge instructions, facilitated follow-up appointments, and provided medication education. In two studies, non-nursing coordinators were used. In one, a non-clinical patient advocate administered a patient survey prior to discharge; this was reviewed by the clinician with the goal of closing the gap between expected and planned discharge information.^50^ Notably, non-English speaking patients were excluded. Another study used volunteers to connect ED patients to outpatient clinics and facilitate social services and financial assistance applications, if needed.

Of the four discharge nursing interventions that measured patient outcomes, two of three found a reduced number of ED return visits,^48^,^51^ two of two reported increased understanding of the discharge instructions,^48^,^49^ zero of two found increased new-prescription medication adherence, and one of three found increased post-ED appointment compliance.^49^ Results of the patient advocate intervention showed enhanced understanding of the discharge instructions, while the volunteer intervention resulted in increased post-ED appointment compliance. No studies measured patient satisfaction or any additional patient-centered outcomes.

### Follow-up Assistance

This subgroup included 10 articles, all except one from the US. Study design was primarily quasi-experimental (5/10, 50%), followed by cohort (4/10, 40%) and RCT (1/10, 10%). Full evaluation of the proposed interventions was limited as over half (6/10, 60%) were not full-text articles. Proposed interventions primarily focused on improving connection to and adherence with post-ED discharge outpatient follow-up appointments. Patients were connected to an appointment scheduler who specifically helped them obtain appointments. When compared to patients who were instructed to schedule their own appointments, there were higher rates of confirmed appointments and increased appointment adherence rates. Having a confirmed appointment prior to ED discharge appeared to have the most significant positive effect on follow-up adherence.^52^,^53^

When patients self-scheduled appointments, follow-up rates improved but not to a statistically significant level and did not change overall patient satisfaction. Additionally, follow-up rate improvement was only seen when a scheduled appointment was confirmed prior to discharge rather than the patient self-scheduling from home.^54^ Despite expressing interest in obtaining follow-up, when instructed how to make these appointments, patients did not appear to read these instructions, leading to poor scheduling and follow-up rates.^54^,^55^

Only one study examined improving communication between ED and outpatient clinicians. This study leveraged the electronic health record to facilitate communication of patients’ follow-up needs post-ED discharge. Both primary care and emergency clinicians perceived this as useful, allowing for best practices of standardized processes and closed-loop communication.^56^ In general, however, there was limited evaluation on the effect of proposed interventions on ED use and return visits or hospitalizations. The only study performing this analysis found an almost 50% reduction in subsequent ED use.^52^ No studies followed up long term or examined hospitalization rates.

### Pharmaceutical Intervention

Of the 16 studies examining pharmaceutical interventions, 12 (75%) were conducted in the US. Study design ranged from quasi-experimental (6/16, 38%), RCT (4/16, 25%), and cohort (3/16, 19%) to case control studies (1/16, 7%).

Involvement of ED pharmacists was at the center of many of these studies with five implementing ED pharmacist review of discharge prescriptions and all demonstrating successful prevention of medication errors.^57^–^62^ Four studies included bedside medication education by ED pharmacists, but with more varied results. Of three studies that examined ED return visits as outcomes, two showed no reduction while one led to significant reduction.^63^,^64^ Patient satisfaction did improve; however, one study showed no reduction in return visits.^63^

Some of the pharmaceutical studies (6/16, 38%) focused on classes of medications (ie, opiates or anti-coagulants)^57^,^58^,^65^–^68^ with interventions involving ED pharmacist review of prescriptions and/or patient education on side effects and safety precautions. These studies showed a decrease in medication errors^57^,^58^,^66^ and an increase in patient awareness.^65^,^67^ Only one study examined longer term patient outcomes, demonstrating lower ED-return visits or hospital readmissions after patient education by an ED pharmacist on new prescription dosing and side effects.

Six studies (6/16, 38%) addressed patients’ access to discharge medication. Four interventions provided medications to patients at ED discharge,^65^,^69^–^71^ with variable results. Two demonstrated a decrease in return visits^65^,^70^ while one demonstrated an *increase* in return visits.^71^ The study that found an increased return visit occurred in an ED where clinicians were encouraged to provide in-hand medications to patients enrolled in financial assistance programs, with the hypothesis that these patients with fewer resources at baseline may already be more likely to use the ED as a usual source of care.

Interventions focusing on pharmacists and discharge medication reduced medical errors, improved patient comprehension, and expanded medication access. Using ED pharmacists for future discharge interventions is likely to be successful; however, this comes at an expense, requiring further cost-benefit analysis. Notably, these studies did not consistently examine long-term, patient-centered outcomes such as admissions related to medication errors or medication adverse-reaction rates.

### Patient-centered Education

Of 28 papers and abstracts that examined interventions focused on patient-centered education, 17 (61%) were conducted in the US. Study design was variable, with 12 RCTs (43%) and eight quasi-experimental studies (29%) making up the largest proportion. Seventeen (64%) focused on four primary interventions: standardizing discharge instructions; using the teach-back method; focusing on disease-specific intervention; and tailoring instructions to patients’ needs.

Standardizing discharge instructions involves predeveloped, written content for common ED presentations. The ED is often busy, with competing interests limiting the emergency clinician’s time to write quality instructions and discuss them with the patient.. Standardized, pre-written instructions ensure that discharge content is up to date, evidence based, thorough, and in plain, simple language for patients to understand. This, in theory, ensures quality instructions regardless of the ED environment. Of the seven studies using this approach, four demonstrated an improvement in patient understanding;^72^–^74^ two showed no change, and one described but did not implement the intervention.^75^

Seven studies (7/28, 25%) examined the use of “teach-back” in providing discharge instructions and found improved comprehension and recollection but decreased patient satisfaction. One study showed that patients found this to be “condescending.”^75^ While using “teach-back” may be effective in conveying key information, patient discontent with the ED encounter could have rippling effects on both the patient-clinician relationship and the patient’s likelihood in heeding their discharge instructions. Five studies (5/28, 18%) centered on disease-specific instructions.^76^–^79^ Each study examined different disease foci and outcomes. Although these studies found positive results, the variability of study design made it difficult to assess generalizability.

Four studies (4/28, 14%) tailored discharge instructions to patients’ needs. Two (50%) focused on healthcare literacy^80^,^81^ and one (25%) used cartoons,^82^ both demonstrating improvement in patient comprehension. One (25%) focused on language^83^ but did not measure patient comprehension. Patient-centered education is a frequent focus of discharge interventions, but with varied and inconsistent results. Consideration should be given to patient satisfaction and generalizability in future studies.

### Clinician/Discharger-Centered Education

This subgroup included 17 articles. Most (10/17 59%) were from the US with study designs including quasi-experimental (7/17, 41%), RCTs (3/17, 18%), cross-sectional (2/17, 12%) and cohort (2/17, 12%).

Several studies found inconsistent and varied discharge processes, leading to limited verbal and written quality of instructions.^73^,^84^ Several studies created formalized discharge guidelines.^85^–^89^ Stake-holder feedback from clinicians and patients created consensus^88^–^90^ on inclusion of the most important components: discharge diagnosis; self-care instructions; follow-up planning; and return precautions.^89^ Clinician re-education on quality discharge instructions led to improved patient comprehension, satisfaction, and reduced ED return visits.^73^ While one study found that a structured workflow doubled the time needed for discharge, overall interaction time with clinicians was still less than five minutes.^85^

While several studies proposed a structured discharge process, only two examined implementing a checklist. In these interventions, clinicians received formal instruction on appropriate discharge processes and a comprehensive list of essential components.^85^,^86^ This led to improved clinician adherence and patient comprehension and satisfaction.^85^,^86^ Similar standardization was used to propose a standardized written discharge summary in one study, which found that while this led to clinician adherence, there was overuse of medical terminology and poor follow-up advice, suggesting this method alone is not sufficient for quality discharge instructions.^73^,^91^

Almost none of the studies examined return visits/hospitalizations or outpatient follow-up, limiting understanding of how streamlined discharge processes affect clinical outcomes. The one study examining this found no improvement in patient adherence to follow-up plans.^86^ One study found that patients did not follow up because they did not understand their discharge instructions and either forgot to or felt uncomfortable seeking clarification.^92^

Some studies focused on educating clinicians to provide discharge instructions that are easily understandable. One study examined templates to help tailor the instructions to patients’ reading level,^80^,^93^ which led to a decrease in ED return visits and readmission.^80^ One study showed a persistent gap in appropriate language translation even when using Google Translate for written instructions.^94^ Another used clinician education sessions to empower them to provide similar education to patients. This intervention improved clinician recall and understanding, the rate of patient education on these topics, and patients’ self-reported comprehension and health-related practices.^95^,^96^

## DISCUSSION

Our review included a broad range of study design, methodologies and outcome measures, reflecting both the multidimensional components and impact of ED discharge, as well as a lack of consensus on which patient- and healthcare-centered outcomes best represent discharge quality and effectiveness. This finding contrasts with the extensive literature on post-inpatient^97^,^98^ transitions of care, as well as pediatric ED discharge,^99^–^101^ represented through systematic reviews in a variety of these patient populations.

While the diversity of proposed discharge interventions demonstrates the complexity of factors influencing high-quality ED discharge processes, patterns of sufficiently similar interventions across the 100 studies included inventions that allowed for thematic analysis. Improving the patient’s understanding of discharge instructions was the most frequently examined and demonstrated the greatest impact on patient outcomes. Individual interventions with the greatest impact on improving comprehension included enhanced discharge discussion and education with dedicated personnel (both discharge nurses and pharmacists); a structured discharge “checklist”; and/or ensuring instructions were delivered at an appropriate reading level by using a variety of written and visual modalities. Most of these interventions led to improved patient comprehension of their follow-up plan as well as more positive perceptions of their ED care and discharge experience.

Interventions standardizing or automating written discharge instructions were the most studied, leveraging technology to help standardize this element of the discharge process, as well as improve patient understanding through simplified language or modes of instruction. While some studies found the intervention increased patient comprehension, a critical gap in assessing the impact of these findings is the fact that patient-centered outcomes, such as adherence to discharge instructions, were not concurrently measured. The few studies that did examine these outcomes did not reliably demonstrate post-intervention improvements in these areas, suggesting that patient comprehension alone may not be a comprehensive outcome measure to determine ED discharge intervention efficacy. We do note, however, that a promising correlation was found between confirming follow-up appointments at time of ED discharge and increased appointment attendance and use of a discharge nurse to connect patients to medical and social outpatient resources and decreased ED return visits. While evaluation of outcome measurements is currently limited, these two interventions warrant further exploration, including refinement of processes, scaling, and validation across healthcare systems and patient populations.

The lack of studies examining intervention effects on long-term outcomes is perhaps the most significant gap in post-ED transitions-of-care literature. Most research on post-ED transition interventions has focused on short-term outcomes, such as follow-up attendance and patient satisfaction, typically within days or weeks of discharge. However, critical patient-centered outcomes, such as unplanned ED visits, hospitalizations, adverse events, and long-term morbidity and mortality, are often overlooked. Emergency department transitions are recognized as high-risk periods for adverse health events; without studying these outcomes, it is difficult to assess the full impact of discharge interventions. As previously mentioned, the importance of examining long-term outcomes in improving ED discharge processes is underscored by a key finding in several of the reviewed studies: despite improvements in patient comprehension of instructions, these gains did not always translate into better adherence to those instructions. This discrepancy highlights a critical gap between patients’ self-reported understanding of their care plan and their actual behavior in following it.

While educating patients about their diagnoses, medications, and follow-up care is a fundamental component of discharge interventions, comprehension alone may not be sufficient to drive the behavior changes necessary for improved clinical outcomes. Factors such as lack of social support, limited health literacy, and other social determinants of health can undermine a patient’s ability to act on the information provided at discharge, even if they grasp it conceptually. Future studies should prioritize long-term cohort studies and RCTs that track both short-term effects and long-term outcomes, including hospitalization, adverse events, and mortality over follow-up periods (eg, 30–90 days). Without this analysis we have limited understanding of the broader and perhaps more consequential implications of discharge interventions on long-term patient health, morbidity, and potential mortality. Additionally, examining how patient factors such as age, comorbidities, and social determinants of health interact with long-term outcomes will help tailor interventions to diverse patient populations.

Consideration of long-term outcomes also plays a crucial role in the cost-benefit assessment of ED discharge interventions. Many of the high-impact interventions identified involve adding specialized personnel, such as discharge coordinators and ED pharmacists, to improve medication adherence and patient comprehension. Addition of personnel is a significant cost; however, none of the studies examined the financial costs of these interventions, creating a gap in understanding their economic feasibility and sustainability. This is particularly relevant in healthcare systems with budget constraints or where these roles have not yet been established. And without including long-term outcomes such as fewer ED visits and readmissions, it is difficult to assess whether the benefits of these intervention outweigh the additional costs incurred from hiring and maintaining these roles.

If future cost-effectiveness and outcomes work does not demonstrate significant return of investment for personnel-related interventions, the role of digitally based interventions may have further promise as they can often be scaled up at lower cost. Future research should integrate cost-effectiveness analyses with long-term, patient-centered clinical outcomes to better balance clinical impact and economic considerations. These types of analyses could enable the development of sustainable discharge interventions and wide adoption across diverse healthcare settings.

Likely contributing to the gaps above was a demonstrated lack of consensus on the most effective outcome measures to assess the quality and impact of current ED discharge interventions and how future ED discharge innovations could demonstrate measurable returns on investment through improved patient and population health outcomes. Given this, future research should focus on standardized, patient-centered outcomes that capture both short- and long-term impacts. Short-term patient-centered outcomes might include not only patient comprehension and satisfaction, but also adherence to discharge instructions as measured through medication adherence, follow-up appointment attendance, and rates of ED return visits for related complaints. Long-term patient-centered outcomes should encompass metrics such as 30- to 90-day ED return visits and hospitalizations, clinically significant adverse events (eg, worsening of disease conditions or medication side effects), and mortality.

In addition to these clinical outcomes, it is crucial to examine implementation outcomes such as the feasibility, cost, adoption, and sustainability of proposed interventions to fully assess their efficacy and potential for widespread use. One current limitation is that there are no consensus-based methods or tools to assess either objective outcomes (eg, ED visits, hospital readmissions, medication adherence) or subjective outcomes such as patient comprehension and satisfaction). Overall, standardizing outcome measures and developing consensus-based tools to evaluate these outcomes could provide more consistent and reliable data, allowing for more robust evaluations of the impact of proposed discharge interventions.

The goal of creating feasible and sustainable discharge interventions is also limited because much of the existing research has focused on individual components of the discharge process rather than comprehensive, integrated interventions. While optimizing individual components of the ED discharge is important, and may impact downstream outcomes, ED discharge is a complex, multidimensional, and multifaceted process. The critical lack of studies examining ED discharge in its entirety and the piecemeal approach may overlook the complexity of post-ED transitions, where multiple factors—ranging from medication management to social support—interact to influence patient health. The previously limited scope of discharge evaluation may explain some of the variation in measured effects observed across different studies. Addressing the full spectrum of needs during the discharge process, while ensuring that interventions are practical and resource-efficient, is key to developing interventions that are not only clinically effective but also feasible and scalable.

Finally, our review revealed a critical gap in studies including non-English speaking patients, who are a vulnerable ED population. Most studies had non-English as an exclusion criterion, especially when measuring patient understanding of discharge instructions, and the perspectives of low-English proficiency (LEP) patients is particularly under-represented in ED discharge improvements.^102^,^103^ The LEP patient faces additional communication barriers, reporting poor understanding of their care,^104^–^106^ which led to clinically significant adverse safety events^4^,^14^ with missed medications and follow-up appointments and higher likelihood of unplanned ED revisits.^11^,^16^ A relatively small number of studies focused on other vulnerable populations such as older adults and patients with lower socioeconomic status. Older age is associated with elevated medication and follow-up appointment nonadherence and higher discharge failure risk.^107^,^108^ A greater burden of comorbid illness and higher prevalence of cognitive impairment already increases non-comprehension risk. By not including these patient populations, this significant gap in discharge process improvement risks disproportionately affecting our most vulnerable patients. Diverse representation is essential as attempts to improve healthcare processes without representative patient involvement means innovations fail to reflect the realities of these patients’ experience.^109^,^110^

## LIMITATIONS

There are limitations to our study and its applicability. We identified only 100 studies examining discharge interventions in adult ED patients. The majority were retrospective cohort studies with few RCTs or prospective study designs and high variability of methodologies and outcome measures. Consequently, systematic review or meta-analysis methodology was not possible. As this was a scoping review that included articles representing a diverse range of methodologies, we did not conduct a formal assessment of the included article and abstract quality or risk of bias, limiting reliability of the studies’ findings. Our review was conducted to include both published and gray literature; however, there may be effective discharge interventions used in EDs that have not been described or publicized in the literature.

For pragmatic reasons, our inclusion criteria also limited the scope to studies written in English, which could potentially have excluded relevant global contributions as well as restricted the generalizability of our findings to global audiences and environments. Furthermore, the review was limited to studies published from 2000 onward, which might have excluded some potentially relevant older interventions and practices.

## CONCLUSION

The EM community has long recognized ED discharge as a complicated and critical aspect of the unscheduled, acute care episode. However, significant gaps in current research methodology exist, including limited prospective studies, lack of consensus on appropriate ED-discharge quality metrics and outcome measures, absence of long-term outcome studies, and scarcity of studies examining the discharge process as a whole, particularly in vulnerable populations. This review identifies some of the promising directions and opportunities for patient-centered innovation, but in comparison to prehospital or inpatient care, innovation and associated research to better understand how to optimally close the adult ED encounter is limited and warrants a call to action. With recent improvements in digital technologies to engage and communicate with patients and across healthcare systems, as well as the appropriate focus on health equity for disproportionately vulnerable ED populations, the time is now. More comprehensive and longitudinal innovations to optimize adult ED discharge processes, with a focus on rigorous evaluations of their impact with standardized metrics, could yield significant improvements in post-ED patient-centered outcomes.

## Supplementary Information



## Figures and Tables

**Figure:**
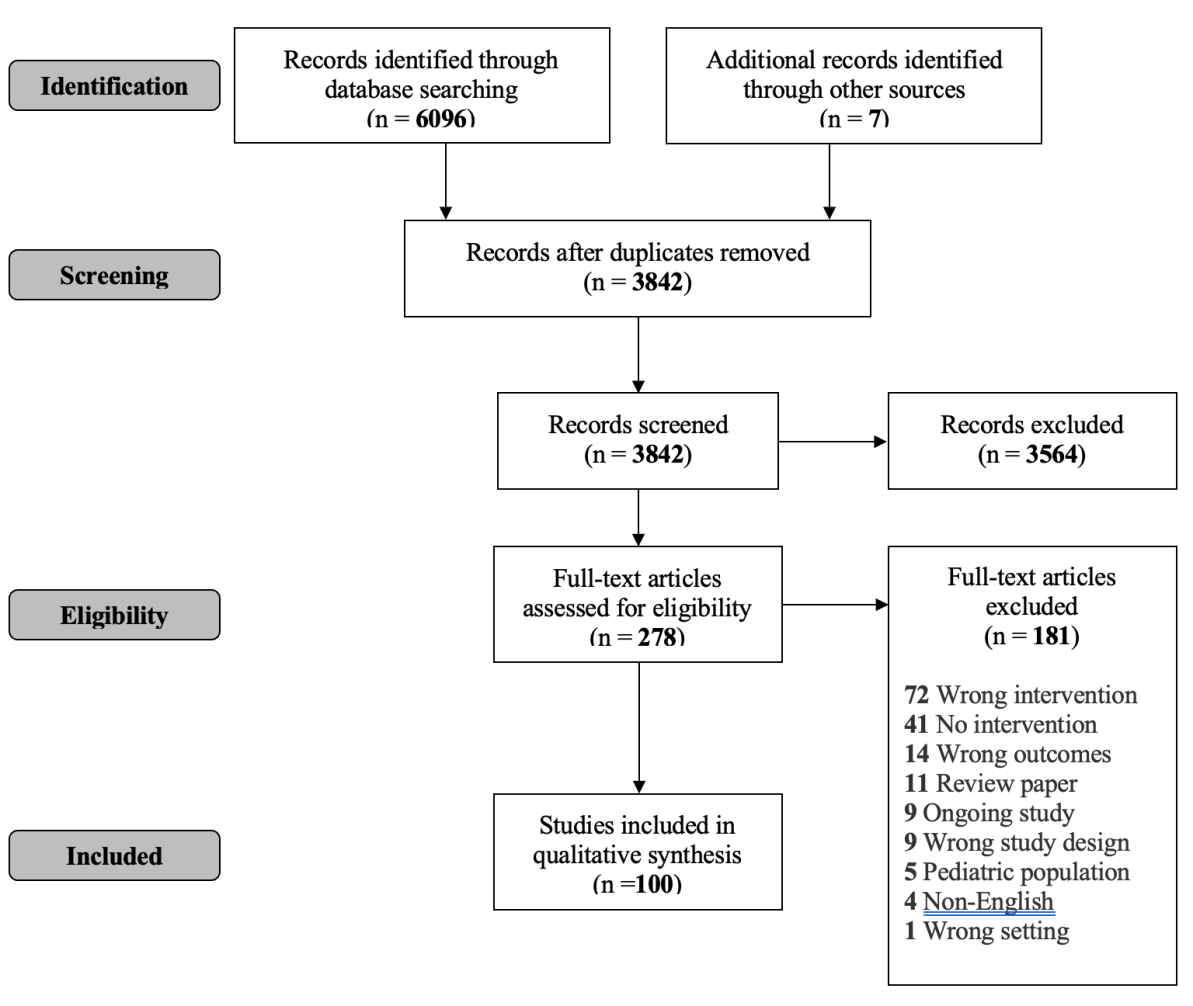
PRISMA* flowchart. *PRISMA, Preferred Reporting Items for Systematic reviews and Meta-Analyses.

**Table 1:** Descriptions of discharge intervention subgroups.

Subgroup title	Subgroup description
Mode of discharge	Interventions focusing on the various methods of delivering discharge instructions
Additional resource provision	Adding social resources and services, either trained medical professionals or volunteers, to the discharge process
Addition of discharge coordinator	Repurposing ED staff or hiring new employees whose primary role is to perform ED discharges
Follow-up assistance	Facilitating the scheduling of outpatient follow-up appointments with primary care and/or specialty clinicians
Pharmaceutical intervention	Interventions focusing on discharge prescriptions
Patient-centered education	Altering the discharge process to improve patient understanding and comprehension of discharge instructions and/or diagnoses
Clinician/discharger-centered education	Interventions centered on teaching and educating clinicians/dischargers on best practices of delivering discharge instructions

*ED, e*mergency department.
