# Supplementary material for: Scoping Review of Adult Emergency Department Discharge Interventions
Source: West J Emerg Med. 2025 Jul 13;26(4):823–34. doi: 10.5811/westjem.35264 (PMC12342426; doi:10.5811/westjem.35264)
Supplement: Supplementary file 1 [file wjem-26-823-s001.pdf]

Name	Study Design	Full Text Paper?	Country	Intervention	Intervention implemented?	*If yes to intervention:* # of subjects	*If yes to intervention:* comparison	*If yes to intervention:* outcomes/results	Subgroup
A new model of care for the discharged emergency department (ED) patient...2009 CENA International Conference for Emergency Nursing	quasi-experimental	No	Australia	Nursing provides relevant, standardized information to patients at time of discharge including referrals if needed	Yes			Allows for more effective communication, and allows patients to discuss their needs with staff. No data seen on return rates	addition of discharge coordinator and/or team
An emergency department-based nurse discharge coordinator for elder patients: does it make a difference? [corrected] [published erratum appears in ACAD EMERG MED 2005 Jan;12(1):12].	quasi-experimental	Yes	Canada	An ED based nurse discharge coordinator, including patient education, coordination of appointments, telephone follow up, and access to the nurses for 7 days after discharge	Yes	819	control	Reduced ED return visits, faciliitates transition to home and into community health	addition of discharge coordinator and/or team
Does a patient advocate during the discharge process improve patient safety and satisfaction in the emergency department?	RCT	No	USA	Research assistant asked patients 5 standard questions about their perceived readiness for discharge, brought concerns to ED Physician's attention	Yes	120	control	Increased the discharge satisfaction in pt's who received a patient advocate	addition of discharge coordinator and/or team
Effectiveness of an advanced practice emergency nurse role in a minor injuries unit.	RCT	Yes	Australia	Nurse focused on pre-discharge care and transition home	Yes	231	Control	More likely to receive written discharge information, info on equipment, meds, and have follow up arranged. Had a better understanding of their condition as well	addition of discharge coordinator and/or team
Implementation of a nursing led intervention bundle in the Emergency Department : Outcomes	cohort	No	Singapore	nurse-led asthma care bundle	Yes	23420	longitudinal over program lifespan	Increased asthma nurse counseling, ICS/ICS/LABA prescription, reduced 30d revisit	addition of discharge coordinator and/or team

The effect of a transitional care nursing intervention to reduce emergency department revisits in a tertiary cardiac hospital: A randomized controlled trial	RCT	No	Canada	3 nurse-patient encounters using standardized evalaute tools to help with clinical issues prior to discharge	Yes	265	control	No significant effect on ED return visits	addition of discharge coordinator and/or team
Impact of a novel volunteer-run discharge planning program on follow-up appointment adherence	quasi-experimental	No	USA	Volunteer college students connecting patients to outpatient clinic appointments	Yes	141	pre/post	nonsignificant increase in follow up adppt adherence	addition of discharge coordinator and/or team; follow up assistance
Evaluating the Feasibility of an Emergency Department Physical Therapy Intervention for Acute Low Back Pain	quasi-experimental	Yes	USA	PT eval, 3 home exercises, return visit in 1 week to evaluate home exercises and symptoms	Yes	30	No comparison	20/27 patients able to demonstrate proficiency in exercises, performed 3 times at home. F	additional resource provision
Geriatric nursing assessment and intervention in an emergency department: a pilot study.		Yes	Denmark	0, 1, 6 mos nursing assessment vs. usual care	Yes	150	Usual care	Intervention group had less unresolved problems and higher utilization of home care services	additional resource provision
Patient-Centered Outcomes of an Emergency Department Social and Medical Resource Intervention.	cross-sectional	Yes	USA	patients provided with optional medical & social resources and all received an education intervention (med list & follow up appt review and emphasis)	Yes	494	no resources requested	Patients who requested both medical & social resources were associated with lower odds ratio of ED revisit at 30 days compared to those that requested no resources (OR 0.5, CI 0.27 - 0.95)	additional resource provision
Discharge Communication in Patients Presenting to the Emergency Department With Chest Pain: Defining the Ideal Content.	cross-sectional	Yes	Switzerland	Mnemonic techniques to improve patient recall	No				patient-centered education; provider/discharger-centered education

136 Can Conventional Discharge Instructions Engage Patients in a Post-Emergency Department Visit Telemedicine Follow-Up Program?	cohort	No	USA	Written instructions about telemedicine follow up	Yes	122	NA	Despite written discharge instructions, patients still reported lack of knowledge of telemedicine follow up program	follow up assistance
Implementation of a novel emergency department discharge center	quasi-experimental	No	USA	Discharge center to help pt's understand discharge and with follow up needs/referrals	Yes	1206	pre/post	Breakdown of ED referrals, inc in referrals from 1.6-> 6.5%, inc patient phone calls, no increase LOS	follow up assistance
Implementation of an electronic medical record based transition of care program from the emergency room to gastroenterology clinics improves followup	cohort	No	USA	Electronic transition of care to direct GI follow-up	Yes	233 + 942	usual GI referral	GI-TOC (vs. pre-TOC program) were more likely to get endoscopies	follow up assistance
Improving Outpatient Follow-Up Through Innovative Appointment Scheduling at Emergency Department Discharge.	quasi-experimental	Yes	USA	Patient access rep trained to help provide PMD and specialty appts at time of discharge	Yes	228	pre/post	Reduction in ED revisit rate for similar or same complaint	follow up assistance
Improving Post-Discharge Follow-up Rates in Trauma Patients: A Doctor of Nursing Practice Project.	cohort	Yes	USA	Re-engineered discharge kit, reminder about follow up appt, and transportation planning home	Yes	53	control	Follow up appointments had increased in attendance rate, but no improvement in hospital readmission rates	follow up assistance
Improving specialty follow-up care after an emergency department visit using a unique referral system	quasi-experimental	No	USA	implementation of patients calling a scheduler and obtaining a specialty appt prior to discharge from the ED for all urgent referrals	Yes	1174	pre/post	Increased compliance rates of follow up appts in specialty clinics	follow up assistance

Randomized controlled trial to improve primary care follow-up among emergency department patients.	RCT	Yes	USA	PCP appt booked through website prior to ED discharge vs. written info on how to use booking website vs. usual care	Yes	272	usual care	follow up rate was improved for pts with appt booked through website 52% vs. those who received booking website info 25% vs. usual care 36%. 78% in booking group reported being extremely or very satisfied 78% compared to those who received booking website info 54% vs. usual care 40%	follow up assistance
The ED-PACT Tool Initiative: Communicating Veterans' Care Needs After Emergency Department Visits.	quasi-experimental	Yes	USA	Tool to send message from ED provider to outpatient PMD for specific follow up needs	Yes	4899	pre/post	Improves communication with outpatient providers	follow up assistance
Does the modality of emergency Department discharge instructions improve patient follow-up with a family physician?	cohort	No	Canada	ED discharge instructions, verbal vs verbal and written vs verbal/written and copy of discharge summary given and sent to PMD	Yes	82, 61, 89 in group 1, 2, 3	other groups	PMD follow up did not change significantly despite mode of discharge	follow up assistance; mode of discharge
130 Patient Preference for Receiving Their Discharge Instructions From the Emergency Department.	RCT	No	USA	Discharge instructions by written text or by video	Yes	196	control	Most patients preferred either video or video plus writtent instructions	mode of discharge
A QIP TO ADDRESS PATIENT-CENTRED CARE BY INCREASING THE DISTRIBUTION OF PATIENT INFORMATION LEAFLETS (PILS) BY 10%, AT THE POINT OF PATIENT DISCHARGE AT PILGRIM HOSPITAL ED, BY MARCH 2021		No	UK	Provide patient info leaflets	Yes			8% increase in rate of giving DCIs to patients	mode of discharge

Awareness of diagnosis and follow up care after discharge from the Emergency Department.	quasi-experimental	Yes	Australia	implementing simplified discharge info card in addition to current care	Yes	221	usual care	awareness of discharge diagnosis improved from 73.2% to 89.7% (CI 82.9 - 94.0)	mode of discharge
Brief educational video plus telecare to enhance recovery for older emergency department patients with acute musculoskeletal pain: study protocol for the BETTER randomized controlled trial.	RCT	Yes	USA	interactive education video about pain meds & recovvery-prompting behaviors + telecare phone call after discharge & electronic communication to pt's PCP vs. video only vs. usual care	No		video + follow up and communication with PCP vs. video vs. usual care		mode of discharge
Can Videoclips Improve Doctor-patients Communication at the Emergency Department? A Randomized Controlled Trial	RCT	No	Italy	Video instructions specific to diagnosis at time of discharge	Yes	Ongoing	Control		mode of discharge
Cognitive Rest and Graduated Return to Usual Activities Versus Usual Care for Mild Traumatic Brain Injury: A Randomized Controlled Trial of Emergency Department Discharge Instructions.	RCT	Yes	Canada	patients with mild TBI discharged from ED received cognitive rest and graduated return to usual activity discharge instructions	Yes	118	usual care	no difference in post-concussion symptom score . the number of follow up appts and time off work/school were similar	mode of discharge
Does the availability of preformatted text in discharge instructions result in improved rates of holding metformin in emergency department patients receiving an IV contrast CT study?	quasi-experimental	No	USA	Insertion of preformatted text to hold Metformin and get renal function rechecked after IV contrast	Yes	99	Pre/post	Despite the availability of the text, providers did not insert this into the discharge instructions	mode of discharge

Effect of personalised, mobile-accessible discharge instructions for patients leaving the emergency department: A randomised controlled trial.	RCT	Yes	Australia	patients discharged from ED with MSK pain received usual pain relief advice with addition of personalized printed & mobile-acessible discharge instructions	Yes	80	usual care	no statstically significant difference in pain scores or global improvement scores. intervention group had higher odds of being "very satisfied" (OR 7.14 CI 1.18-50) & hiigher recall of pain meds advice (OR 20, CI 1.56-100)	mode of discharge
Effect of standardized electronic discharge instructions on post-discharge hospital utilization.	quasi-experimental	Yes	USA	templated discharge instructions vs. usual care	Yes	16572+17516	usual care	no significant changes	mode of discharge
Emergency Department Discharge Instructions: Lessons Learned through Developing New Patient Education Materials.		Yes	USA	Specifically tailored discharge instructions for a diagnosis	No				mode of discharge
Empowering patients: simplifying discharge instructions.	quasi-experimental	Yes	USA	development of one-page simnplified information page targeted to inform patients of their most relevated discharge instructions	Yes	236	usual care	increase in intervention group's discharnge understanding with an increase of questionnaire score by 22%	mode of discharge
Establishing a written advice sheet to patients consulting for wound to emergency ward improves postemergency care.	quasi-experimental	Yes	France	implementation of wound care advice sheet based on consensus conference recommendations	Yes	119	usual care	no difference in antiseptic or soap use, wound complications. intervention group had 91.7% adherence to wound care instructions vs. 72.9% (p = 0.02), lower dressing changes 5.45 vs. 12.9 (p = 0.001) & suture removal date was more in agreement with recommendations 83.9% vs. 66.7% (p = 0.03)	mode of discharge

Evaluating Patient Motivation and the Use of Online Health Information: Keeping Patients and Families in the Loop.	cross-sectional	Yes	USA	Evaluation of understanding of discharge instructions, motivation, and the use of online resources	Yes	100		Patients did not understand their discharge instructions, would be open to using online resources more commonly, and ability to understand through audio or video resources may increase understanding of discharge instructions	mode of discharge
Guidelines for discharge: do standardized cards help in patient understanding?.	RCT	Yes	Brazil	Standardized discharge instruction cards vs. routine care	Yes	228	routine care	Standardized card patients followed more instructions than routine care (p=0.009)	mode of discharge
Improvement of Communication with Primary Care Practitioners with The Use of Emergency Department Discharge Summaries.	quasi-experimental	Yes	Ireland	Introduction of ED discharge diagnoses and summaries to be provided to PCP	Yes	50	Control	Introduction of eDischarge summaries improved quality and safety of the ED discharge process	mode of discharge
Information technology improves Emergency Department patient discharge instructions completeness and performance on a national quality measure: a quasi-experimental study.	quasi-experimental	Yes	USA	Electronic discharge instructions vs. paper-based	Yes	300	Electronic vs paper	97.3% OP-19 compliance for digital vs. 46.7% for paper	mode of discharge
Minor head injury in the Republic of Ireland: evaluation of written information given at discharge from emergency departments.	quasi-experimental	Yes	UK	Evaluation of existing information given at discharge to patients with minor head injuries				Discharge instructions were brief, had variable advice for symptom management, were not standardized, and omitted important symptoms	mode of discharge

MyEDCare: Evaluation of a Smartphone-Based Emergency Department Discharge Process.	quasi-experimental	Yes	USA	paperless, smartphone-based discharge process initiated	Yes	16933	usual care	intervention group had less frequent 72-h unscheduled return (3% vs. 5.6% p = < 0.001), unscheduled 9-d return (6% vs. 10.3% p = < 0.001), and unscheduled 30-d return (10.7% vs. 16.9% p = < 0.001)	mode of discharge
Randomized pilot trial measuring knowledge acquisition of opioid education in emergency department patients using a novel media platform.	RCT	Yes	USA	educational Khan Academy-style animation discharge instruction on dangers & safe use of opioid analgesics	Yes	52	usual care	intervention group scored 82% on knowledge acquisition test vs. 65%	mode of discharge
Seventy-two-hour antibiotic retrieval from the ED: a randomized controlled trial of discharge instructional modality.	RCT	Yes	USA	Standard Discharge Instructions alone vs. SDIs + text message vs. SDIs + VM instructions	Yes	2521	Standard Discharge Instructions alone	1. Nonsignificant effect on antibiotic retrieval rates overall2. Text message outperformed VM3. VM underperformed control	mode of discharge
Speak fast, use jargon, and don't repeat yourself: a randomized trial assessing the effectiveness of online videos to supplement emergency department discharge instructions.	RCT	Yes	Canada	Online video discharge instructions vs. routine care	Yes	133	Online vs. paper	19% higher mean scores in post instructions testing	mode of discharge
Utilization of Educational Videos to Improve Communication and Discharge Instructions.	quasi-experimental	Yes	USA	patients who were discharged from the ED with one of 5 selected discharge diagnoses received written discharge instructions PLUS video discharge instructions	Yes	240	usual care	comprehension questionnarie showed better scores for patients who received video instructions vs. usual care (30.1% vs. 52.3% p < .001)	mode of discharge

Improving patient understanding of emergency department discharge instructions	quasi-experimental	No	USA	pre/post implementation of standard discharge instructions	Yes	45	pre/post	Intervention patients were more likely to score better on a posttest	mode of discharge; patient-centered education
the nosebleed effect': Advice given to epistaxis patients discharged from the emergency department	cohort	No	UK	Advise on bleed prevention and give info on future epistaxis management	No				mode of discharge; provider/discharger-centered education
"Like a dialogue": Teach-back in the emergency department.	cross-sectional	Yes	USA	Teach-back	Yes	51	NA	Participants thought teach-back would help, but voiced concerns that it may come across as condescending.	patient-centered education
A proposal for an evidenced-based emergency department discharge form for mild traumatic brain injury.		Yes	USA	standard discharge forms using evidence-based core segments	No				patient-centered education
A randomized controlled trial of a comprehensive migraine intervention prior to discharge from an emergency department.	RCT	Yes	USA	Extra instructions + pills in pocket + expedited neuro follow-up vs. routine care	Yes	50	Usual care	No effect on HIT-6 disease imapct surey score, but higher rates of initiation/adherence to triptans or other migraine-specific therapy	patient-centered education
Discharge Instructions for Concussion: Are We Meeting the Patient Needs?	RCT	Yes	USA	Booklet on concussion instructions put together using current guidelines and home instructions	Yes	12	Regular discharge instructions	No increase in knowledge or preference of using the booklet vs standard concussion discharge instructions	patient-centered education
Facilitating anticoagulation for safer transitions: preliminary outcomes from an emergency department deep vein thrombosis discharge program.	cohort	Yes	USA	FAST program for transition to outpatient anticoagualation	Yes	32	Na		patient-centered education

Information structuring improves recall of emergency discharge information: a randomized clinical trial.	RCT	Yes	Switzerland	different groups of students were tested for information recall after structured vs. non structured presentation of inofrmation	Yes	234		structured discharge info led to relative increase in recalled items (17%) in all levels of medical knowledge. in the sample with the least medical knowledge, structured discharge info resutled in relative increase by 42%	patient-centered education
Long-term benefits of education by emergency care nurses at discharge of patients with atrial fibrillation.	quasi-experimental	Yes	Spain	atrial fibrillation teaching by emergency medicine nurses	Yes	240	usual care	AF-related or treatment-related complications and death were lower in interention group (31.9% vs. 48.4%, p = 0.005)	patient-centered education
Patient comprehension of emergency department care and instructions: are patients aware of when they do not understand?.	cross-sectional	Yes	USA	test patients immediately after discharge instruction delivery (e.g. teachback) to ensure they're not falsely confident	No				patient-centered education
Patient input into the development and enhancement of ED discharge instructions: a focus group study.		Yes	USA	Patients should be involved in creating discharge instructions	No				patient-centered education
Quality Improvement: Using Teach-Back to Improve Patient Satisfaction during Discharge in the Emergency Department.	quasi-experimental	Yes	USA	emergency nurses underwent training sessions and received emails regarding using the teach-back method when discharging patients	Yes	7264	usual care	post-intevention more patients answered "yes definitely" to the question "did the care providers explain what to do if you did not get better after leaving?" from 59% to 61%	patient-centered education
Recall of discharge advice given to patients with minor head injury presenting to a Singapore emergency department.			Singapore	Verbal vs. printed DCIs	Yes	110	verbal vs. printed	No significant difference	patient-centered education

Simple educational intervention to improve the recovery from acute whiplash: results of a randomized, controlled trial.	RCT	Yes	Canada	educational pamphlet vs. usual care	Yes	112	usual care	no significant effect	patient-centered education
Standard versus patient-centred asthma education in the emergency department: a randomised study.	RCT	Yes	Australia	patient centered asthma education vs. routine care	Yes	146	usual care	revisit was 22% vs. 12% in intervention group	patient-centered education
Teach-back of discharge instructions in the emergency department: a pre-post pilot evaluation.	quasi-experimental	Yes	Netherlands	ED nurses, physicians and residents received teach-back training via newsletter, posters and info cards. Training sessions conducted as well with role play	Yes	648	usual care	ED revisits within 7 days were lower (AOR of 0.23 CI 0.05 - 1.07) and 8-30 days (AOR 0.42 CI 0.14 -1.33). Intervention group had increased likelihood of full knowledge retention on info related to ED diagnosis and treatment (AOR 2.15, CI 1.01 - 4.75), medication (AOR 14.89 CI 4.12 - 53.85) & follow up appts (AOR 3.86 CI 1.33 - 10.19)	patient-centered education
The effectiveness of a health education intervention on self-care of traumatic wounds.	quasi-experimental	Yes	Taiwan	specific 25 minute wound care program vs. usual care	Yes	89	usual care	Patient post-test questionnaire responses and satisfaction were significantly higher after program	patient-centered education
The Impact of Teach-Back Method on Retention of Key Domains of Emergency Department Discharge Instructions.	quasi-experimental	Yes	USA	addition of teach-back method to standard discharge instructions	Yes	209	usual care	recall rate was improved in intervention group (82.1% vs. 70.0%, $p < 0.005$)	patient-centered education

The impact of teach-back on comprehension of discharge instructions and satisfaction among emergency patients with limited health literacy: A randomized, controlled study.	RCT	Yes	USA	Teachback vs. usual care	Yes	408	usual care	Teachback improves comprehension but not satisfaction or perceived comprehension	patient-centered education
The impact of teach-back on patient recall and understanding of discharge information in the emergency department: the Emergency Teach-Back (EM-TeBa) study.	cohort	Yes	Netherlands	instructed nurses to use "teach-back method" when discharging via presentations, letters and pocket cards	Yes	483	usual care	improvement in scores in 4 domains: diagnosis, treatament, follow-up consultation, return precautions (mean total difference 0.23 with CI of 0.29 - 0.18). proportion of patients with comprehension deficits in at least one of 4 domains decreased from 49% to 11.9%	patient-centered education
Use of teachback discharge instructions does not improve patient satisfaction in the ED	RCT	No	USA	Use of teach back discharge instructions	Yes	122	control	Teachback did not improve patient satisfaction rates	patient-centered education
Using health literacy and learning style preferences to optimize the delivery of health information.	RCT	Yes	USA	1. Extra health-literacy-tailored instructions for HTN vs. usual care<br>2. Extra health-literacy+learning style-tailored instructions vs. usual care	Yes	85, 87	usual care	accounting for health literacy and learning style outperforms accounting for health literacy only	patient-centered education
Using Written Instructions to Improve the Quality of Emergency Department Discharge Communication: An Interdisciplinary, Patient-Centered Approach.	quasi-experimental	No	Canada	development of written hand outs for the top 10 ED diagnoses	Yes	1001	usual care	improvement in patients receiving written discharge instructions (9.2% vs. 46% p < .001), proportion of patients understanding new symptoms increased from 70% to 84% (p < .0001). 97% of respondents felt discharge handouts were helpful	patient-centered education

Does physician education and factsheet impact on safe opioid use in emergency patients?	RCT	Yes	Australia	ED physician given an education session on safe use of opioids for acute painful conditions and asked to provide education and distribute factsheets to patients	Yes	123	Control. no ED physician intervention	More likely to recall dosag and adverse effects. Improved practices of storage, driving, co-ingestion	patient-centered education; provider/discharger-centered education
Using Health Literacy to Improve Emergency Department Discharge.	RCT	Yes	USA	Instructions written to health literacy levels, and tailored to specific learning style plus health literacy levels	Yes	85, 87	Control	Personalized instructions tailored to learning style and health literacy levels improved pt understanding of htn	patient-centered education; provider/discharger-centered education
"medications in hand" to improve clinician and patient comfort with emergency department discharge	quasi-experimental	No	USA	Pt's with barriers to care/meds, given full course of medications by ED pharmacist for specific medical conditions	Yes	595	Pre/post	119 averted admissions, 9% reduction in 60-day ED return visit when compared to pre-intervention data	pharmaceutical intervention
A Multifaceted Intervention to Improve Patient Knowledge and Safe Use of Opioids: Results of the ED EMC2 Randomized Controlled Trial.	RCT	Yes	USA	Pts discharged from the ED with new norco prescription received an electronic medication complete communication (EMC) + text message vs. just EMC vs. usual care	Yes	343	usual care	demonstrated safe opioid use occurred more often in EMC group (OR 2.46 1.19 - 5.06) compared to usual care, but not EMC + group (OR 1.87, CI 0.90 - 3.90). Neither intervention arm imrpoved med safe use as measured by med diary data. Med knowledge was greater in EMC + text group vs. usual care	pharmaceutical intervention
Discharge prescription optimization by emergency medicine pharmacists in an academic emergency department in the United States.	cohort	Yes	USA	EM pharmacists receiving alert of new discharge prescription and reviewing it	Yes	4575	no pharmacy review	EM pharmacists provided drug recommendations in 7.3% of discharged patients, with 29% rated as clinically significant	pharmaceutical intervention

Does providing prescription information or services improve medication adherence among patients discharged from the emergency department? A randomized controlled trial.	RCT	Yes	USA	Usual care vs.:1. services to reduce barriers to prescription filling2. consumer drug information from MedlinePlus3. both	Yes	3940	routine care	At one of the three 3 ERs tested, all intervention groups had higher prescription fill rates.	pharmaceutical intervention
Impact of a Pharmacist Intervention in the Emergency Department on the Appropriateness of Direct Oral Anticoagulants Prescribed in Venous Thromboembolism Patients.	cohort	Yes	USA	orderset developed for anticoagulation in pts with VTE, which activated an automated alert to the pharmacy in-basket. Pharmacist would then asssess patient and leave progress note with recommendations and counsel patient on the medication	Yes	58	usual care	rate of medication errors was lower when a pharmacist was involved 7.1% vs. 36.4% ($p = 0.046$)	pharmaceutical intervention
Impact of Discharge Anticoagulation Education by Emergency Department Pharmacists at a Tertiary Academic Medical Center.	cohort	Yes	USA	patients discharged with new anticoagulation prescription received education from an ED pharmacist when on site	Yes	174	usual care	patients who did not receive pharmacist education prior to discharge required an increased need for intevention during callback vs. those who did not (36.4% vs. 12.9% $p = 0.0005$). patients who didnt receive intervention were more likely to be readmitted to a hospital or return to ED within 90 days for anticoagulation-related problem (12.12% vs. 1.85% $p = 0.0069$)	pharmaceutical intervention

Implementation of an emergency department-based clinical pharmacist transitions-of-care program.		Yes	USA	pharmacists provide patient-specific comprehensive medication review and education for pts presenting with asthma, COPD & CHF. Referral made to ambulatory care pharmacy clinic or home-bsed medication management	Yes	18		27.7% referred for follow up were successfulo in follow up	pharmaceutical intervention
Implementation of pharmacist targeted discharge prescription review in an emergency department.	quasi-experimental	Yes	USA	EM Pharmacists reviewing discharge scripts, discussing with providers, or helping adjust scripts	Yes	378	Control	EMP's intervened in 18% of discharge prescriptions	pharmaceutical intervention
Improving patient knowledge and safe use of opioids: a randomized controlled trial.	RCT	Yes	USA	Specific instructions about opiates	Yes	274	Routine instructions (no specific pharm instructions)	Intervention group knew more precautions for taking too much tylenol, and were less likely to drive after taking hydrocodone, and were more likely to know that opiates are addictive	pharmaceutical intervention
Improving the safety of anticoagulation initiation in patients discharged from the emergency department.	quasi-experimental	Yes	Australia	patients discharged on anti-coagulation had mandated pharmacy review with real-time EMR alert and automated email sent to ED pharmacists	Yes	491	usual care	84.5% anticoagulant prescriptions reviewed by pharmacist pre-intervention vs. 99.6% post intervention. Of the prescriptions not reviewed by pharmacist, 52.6% had safe initiation while random sampling of those reviewed by pharmacist had 100%.	pharmaceutical intervention
Initiation of a discharge pharmacy within a busy urban emergency department: The first year.		Yes	USA	opened a discharge pharmacy within the adult ED serving only patients discharged from the ED	Yes	5703	NA	35.4% of prescription were considered high-risk, >50% of the high risk meds were purchased through reduced cash pricing, pharmacist interventions were made on 4.3% of prescriptions	pharmaceutical intervention

Outpatient management of deep vein thrombosis using direct oral anticoagulants is safe and efficient.	case-control	Yes	Australia	Rivaroxaban discharge pack	Yes	120		All patients were safely prescribed the medication, with decrease in hospitalizations	pharmaceutical intervention
Patients given take home medications instead of paper prescriptions are more likely to return to emergency department	RCT	Yes	USA	Either given take home med pack or standard prescription	Yes	268	standard prescription	Patients who received take home med pack had an all cause higher return rate to the ED	pharmaceutical intervention
Pharmacist delivered discharge medication counseling in the emergency department and effect on 30-day emergency department and hospital reutilization rates	quasi-experimental	No	USA	Pharmacist delivered medication counseling upon discharge	Yes	65	No counseling	Did not result in decrease ED return rate	pharmaceutical intervention
Protocolised medication after discharge from an emergency department	quasi-experimental	No	Spain	Packets of medications dispensed by ED physician for 5 most common diagnoses	Yes	4320	pre/post	Saving in money for pharmacy, ability to treat more patients	pharmaceutical intervention
The effectiveness of pharmacist- led discharge medication counselling in the emergency department (ExPLAIN): A pilot quasi-experimental study.	quasi-experimental	Yes	Australia	Patients discharged from the ER withnew medications received medication counselling using structured, multimodal educational strategy with teach-back, led by pharmacists	Yes	51	usual care	patients who received intervention had higher satisfaction (p = 0.009). intervention was associted with 98% increase in satisfaction with info. related to side effects. no differences in re-presentaiton and length of stay	pharmaceutical intervention
A Quality Improvement Project to Improve Education Provided by Nurses to ED Patients Prescribed Opioid Analgesics at Discharge.	quasi-experimental	Yes	USA	nurses trained on opiod safety and patient education and then delivered dual-modal (Verbal and written) education with teach-back	Yes	36	usual care	nurses who were trained had an increase in opioid knowledge (73% vs. 98%). 100% of patients clearly understood how to take their pain medications	provider/discharger-centered education

Assessing Documentation of Critical Imaging Result Follow-up Recommendations in Emergency Department Discharge Instructions.	quasi-experimental	Yes	USA	implementation of an electronic discharge module that enabled providers to include follow up recs in the dc instructions	Yes	240	usual care	follow up recs increased from 60% to 73.8% (p = 0.03), no significant change in rate of documented critical imaging findings	provider/discharger-centered education
Assessing the Use of Google Translate for Spanish and Chinese Translations of Emergency Department Discharge Instructions.		Yes	USA	Use google translate for Spanish and Chinese translation	Yes			2% of Spanish and 8% of Chinese sentence translations had potential for significant harm. Overall content was inaccurate owing to grammar or typographical errors that would readily have been overlooked or understood by a reader of the English text	provider/discharger-centered education
Compliance with emergency department discharge instructions.	cross-sectional	Yes	France	HCP training to improve their discharge instruction dispensation is important	No	36			provider/discharger-centered education
Development of a patient-centred, evidence-based and consensus-based discharge care bundle for patients with acute exacerbation of chronic obstructive pulmonary disease.		Yes	Canada	development of COPD discharge bundle based on clinicain and patient input	No				provider/discharger-centered education
Development of the Uncertainty Communication Checklist: A Patient-Centered Approach to Patient Discharge From the Emergency Department.		Yes	USA	Development of checklist to assess uncertainty during discharge communication	No				provider/discharger-centered education
Does a Standardized Discharge Communication Tool Improve Resident Performance and Overall Patient Satisfaction?.	cohort	Yes	USA	Resident education and use of a standard discharge tool, evaluated by attendings	Yes	400	pre/post	Improves satisfaction, discharge performance, and patient perception	provider/discharger-centered education

Evaluation of the R2D2 protocol: A Novel Method for Assessing Emergency Department Disposition for Residents.	quasi-experimental	Yes	USA	ER residents were trained in "R2D2" discharge protocol	Yes	235	usual care	88% of patients in intervention group described their diagnosis correctly vs. 74% (p-value 0.0062) & 91% of patients in the intervention group described treatment plan correctly vs. 67% (p-value 0.0259)	provider/discharger-centered education
Family involvement in emergency department discharge education for older people.	quasi-experimental	Yes	FInland	Interviews of elder patients, family members, nurses regarding family involvement in discharge	No			Family members not seen as participants in ED care of elder patients	provider/discharger-centered education
Hospitalization and discharge education of emergency department patients with hypoglycemia.	cohort	Yes	USA	national guidelines for hypoglycemia should be stated in discharge instructions	No	636			provider/discharger-centered education
Improving emergency department discharge instruction readability	quasi-experimental	No	USA	Provider education/reminders to write discharge instructions at lower reading levels	Yes	91 DIs	pre/post	reduced reading level after QI	provider/discharger-centered education
Increasing Competence and Comfort: Heart Failure Education for the Emergency Department Nurse.	quasi-experimental	Yes	USA	Educational intervention for RN's regarding providing discharge instructions for Heart failure in the ED	No				provider/discharger-centered education
Pre-formatted written discharge summary-a step towards quality assurance in the emergency department.	RCT	Yes	India	Including lab results on discharge instructions vs. usual care	Yes	200	usual care	quality review of carbon copies of these discharge instructions showed they were meh	provider/discharger-centered education
Registered Nurses and Discharge Planning in a Taiwanese ED.	quasi-experimental	Yes	Taiwan	Obtaining information from nursing about what they believe discharge planning should look like from the ED	Yes	25		Found to be no guidelines on discharge planning in the ED, heavy workload limiting ability to plan discharge, and negative/unfriendly attitude of patients and family	provider/discharger-centered education

Communicating Information to Patients: the use of Cartoon Illustrations to improve comprehension of instructions	RCT	Yes	USA	Wound care instructions w/ cartoon illustrations vs. usual care (instructions w/o cartoons)	Yes	205	usual care	Cartoon instructions were much more likely to be read (98% vs. 79%, $p<0.001$), and recipients were more likely to answer wound care questions correctly (46% vs. 6%, $p<0.001$). They were more likely to report compliance with wound care (77% vs. 54%, $p<0.01$)	patient-centered education
Simplification of Emergency Department Discharge Instructions Improves Patient Comprehension		Yes	USA	Simplified discharge instructions	Yes	423	usual care	Patients answered questions re. their discharge instructions more accurately if they were given simplified discharge instructions	patient-centered education
